# A retrospective review of telehealth services for children referred to a paediatric nephrologist

**DOI:** 10.1186/s12882-015-0127-0

**Published:** 2015-08-01

**Authors:** Peter Trnka, Megan M. White, William D. Renton, Steven J. McTaggart, John R. Burke, Anthony C. Smith

**Affiliations:** Queensland Child and Adolescent Renal Service, Queensland, Australia; Department of Paediatrics and Child Health, The University of Queensland, Brisbane, Australia; Centre for Online Health, The University of Queensland, Brisbane, Australia; Queensland Children’s Medical Research Institute, Brisbane, Australia; Queensland Child and Adolescent Renal Service, Lady Cilento Children’s Hospital, 501 Stanley Street, South Brisbane, Queensland 4101 Australia

**Keywords:** Telemedicine, Telehealth, Paediatric nephrology, Kidney disease, Cost savings

## Abstract

**Background:**

Telemedicine has emerged as an alternative mode of health care delivery over the last decade. To date, there is very limited published information in the field of telehealth and paediatric nephrology. The aim of this study was to review our experience with paediatric telenephrology in Queensland, Australia.

**Methods:**

A retrospective audit of paediatric nephrology telehealth consultations to determine the nature of the telehealth activity, reasons for referral to telehealth, and to compare costs and potential savings of the telehealth service.

**Results:**

During a ten-year period (2004 – 2013), 318 paediatric telenephrology consultations occurred for 168 patients (95 male) with the median age of 8 years (range 3 weeks to 24 years). Congenital anomalies of the kidney and urinary tract (30 %), followed by nephrotic syndrome (16 %), kidney transplant (12 %), and urinary tract infection (9 %) were the most common diagnoses. The estimated cost savings associated with telehealth were $31,837 in 2013 (average saving of $505 per consultation).

**Conclusions:**

Our study suggests that paediatric telenephrology is a viable and economic method for patient assessment and follow up. The benefits include improved access to paediatric nephrology services for patients and their families, educational opportunity for the regional medical teams, and a substantial cost saving for the health care system.

## Background

Queensland is the second largest and third most populous state in Australia with a landmass of almost two million square km and a widely distributed population of 4.7 million people [1]. Approximately half of the population is situated in the southeastern corner around the capital Brisbane, while the remainder of the population reside in smaller rural and regional towns, mostly along the coastline. General health care is provided by rural and regional health care centres, with specialist services located centrally in tertiary hospitals in Brisbane. The majority of subspecialist paediatric health services in Queensland are situated in the south-east region of the state, which means that more than half of the population must travel significant distances to access specialist medical care.

Irrespective of where patients live, face-to-face outpatient appointments with a specialist at a tertiary hospital are the most common mode of health delivery. Occasionally, specialist services are offered in regional hospitals when specialist teams travel to provide outreach clinics. Whilst outreach clinics are an effective way to deliver specialist services into remote communities, they are expensive and only occur intermittently. With the current emphasis on cost-effective health care and financially sound health care models, telemedicine has emerged as an alternative means of health care for patients living in remote and rural communities [2]. Telepaediatric burns service and telepsychiatry are examples of successful implementation of this new model of health care delivery for children in Queensland [3–5].

In November 2000, the Centre for Online Health (COH) at the University of Queensland pioneered the establishment of the Queensland Telepaediatric Service (QTS) at the Royal Children’s Hospital (RCH) in Brisbane, to improve access to specialist paediatric health services through the use of telehealth. Routine telehealth clinics were established for a broad range of paediatric sub-specialties, including nephrology. Specialists were given access to a fully supported telehealth service, including dedicated videoconference studios and experienced telehealth coordinators who manage referrals, provide technical support and assist with the delivery of telehealth services [6]. Through this service, routine videoconference clinics have been made available to 106 regional sites in Queensland. In the first 13 years, the QTS facilitated almost 19,000 consultations for children referred to one of 37 different paediatric specialties [7].

Paediatric nephrology lends itself well to telemedicine because it is a subspecialty which offers a centralised service in Brisbane (including dialysis and transplantation) and supports patients living across a large geographical area. Much of the non-acute work involves dialogue with other clinicians and interpretation of test results. Telehealth referrals for paediatric nephrology cases have been managed by the QTS since the service began in 2001 [6].

The aim of this study was to review paediatric nephrology telehealth consultations in Queensland during a ten year period with the emphasis on the number of consultations, the spectrum of renal diseases and the economic implications of these consultations when compared to standard outpatient visits at the tertiary hospital.

## Patients and methods

This study was a retrospective audit of paediatric nephrology telehealth consultations through the QTS over a 10-year period from January 2004 to December 2013. The study was approved by the Children’s Health Queensland Hospital and Health Service Human Research Ethics Committee.

### Clinic model

Over the last decade, the Queensland Telepaediatric Service (QTS) at the COH has provided telepaediatric services to most regional centres across Queensland for a broad range of paediatric subspecialist services. For the paediatric nephrology department at the RCH, the use of telehealth began with an *ad hoc* basis and intermittent clinics, for a couple of regional hospitals. In time, paediatric nephrology telehealth services have expanded – with the QTS managing scheduled telenephrology clinics for 15 remote sites in Queensland. The clinics are well organized with a QTS coordinator ensuring all participants are aware of the proceedings. All technical functions of the telehealth sessions are managed by the coordinators, allowing the specialists to focus on their engagement with the patients and clinicians at the remote site. Patients are referred to the paediatric nephrology service by the family physician and paediatric teams from various locations across Queensland. Referrals are sent directly to the QTS and the case is allocated to one of the scheduled clinics with a paediatric nephrologist. Two paediatric nephrologists participated in telehealth consultations between January 2004 and January 2011, with the addition of the third nephrologist from February 2011.

### Technical aspects

Consultations were coordinated by and carried out by videoconference through facilities at the COH. Videoconferencing was done using the Queensland Department of Health’s IP network which spans across the state and connects over 300 facilities. The COH and remote sites were equipped with dedicated videoconferencing systems, including TV screens, commercial grade codec, and a pan-tilt-zoom camera. The general connection speeds ranged from 512 kbit/s to 2.3 Mbit/s, depending on site-specific connections. Telehealth consultations were attended by the local referring team (paediatrician ± paediatric trainee, nurse), the central paediatric nephrology team (nephrologist ± nephrology trainee, nurse), and the patients and their families. During each consultation clinical information was recorded in the patient’s medical record, according to the same guidelines used for a face-to-face consultation. On the completion of a telehealth consultation, appointment details (including the length of the consultation and the plan for the follow up appointment) were recorded in the hospital service register and the COH research database.

### Data collection

A list of paediatric nephrology patients seen during the telehealth consultations between January 2004 and December 2013 was obtained from the COH. Identified patient medical records were reviewed and the following data were collected for each patient: name, date of birth, gender, referring site, attending nephrologist, type of the consultation (new, review), diagnosis, outcome of the consultation (follow up telehealth review by nephrologist, or review by the local health care team). For the comparison of the telehealth activity with the standard face-to-face appointment, outpatient activity was obtained from outpatient data stored in an RCH database during the same period (January 2004 – December 2013). No other data were collected for the patients attending face-to-face consultations.

### Cost analysis

To estimate potential cost savings, we compared the estimated costs of the nephology telehealth consultations, offered by the QTS, to the potential costs incurred if the patients had travelled to Brisbane for a standard face-to-face visit. The estimation was based on telehealth activity reported during a 12 month period (January to December 2013). Costs accounted for in this analysis included the costs associated with the provision of telehealth services, staff salaries and travel. Given that the telehealth service and outpatient department were already established, certain fixed costs (such as equipment and infrastructure) were not included in the analysis. The costs associated with travel expenses were calculated assuming all patients would have travelled to the specialist hospital in Brisbane using the rebates that would have been provided through the Patient Travel Subsidy Scheme (PTSS), a subsidy provided by the government to assist patients and carers with travel expenses and accommodation costs incurred when a child requires specialist care and must travel further than 50 km. In Queensland, the health department spends around $52 million annually on the PTSS [8]. Accommodation costs for one night were included where air travel was the most appropriate form of transport as it was assumed to be unlikely that a flight could have been booked for return the same day. Additional costs to the family, such as time off work, parking, fuel and meals were not included in this study. Therefore, only direct costs to the health system were included in cost analysis. All costs described in this analysis are reported in Australian Dollars and rounded to the nearest dollar.

## Results

During the ten year study period, 318 paediatric nephrology telehealth consultations were done for 168 patients (95 male, 73 female) with the median age 8 years (range 3 weeks to 24 years). There were 153 new (48 %) and 165 review (52 %) consultations. The number of consultations per patient ranged from 1 (in the majority of patients) to 25 (a patient with posterior urethral valves and a kidney transplant). The average time for a telehealth session was 30 min with allowance for up to one hour, depending on the number of patients booked to the clinic.

### Participating centres

**Fig. 1:**
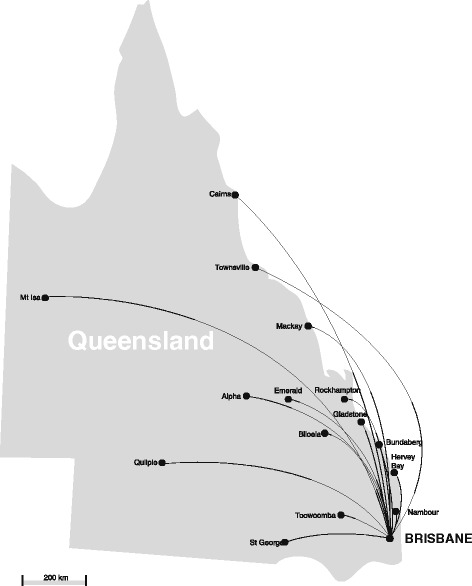
Regional centres participating in paediatric telenephrology in Queensland

Fifteen regional centres across Queensland were involved in the telehealth consultations during the ten-year study period (Fig. 1). The average distance between the COH in Brisbane and the referring site was 868 km, ranging from 125 km (Toowoomba) to 1822 km (Mt Isa). Most referrals originated from Mackay (805 km from the RCH) with 162 (44.6 %) cases during this time, followed by Hervey Bay (287 km from the RCH) with 81 consultations (22.3 %), Rockhampton (636 km from the RCH) with 36 consultations (9.9 %) and Townsville (1357 km from the RCH) with 35 consultations (9.6 %). Thirty-four telehealth consultations (10.7 %) involved multiple sites.

#### Changes in telehealth activity over time

**Fig. 2:**
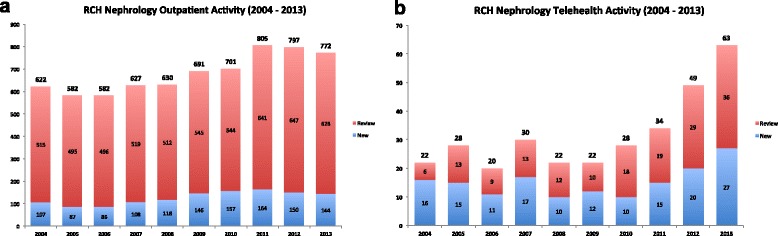
Annual paediatric nephrology outpatient activity in Queensland (2004 – 2013), comparing face-to-face visits (**a**) and telehealth consultations (**b**) at the Royal Children’s Hospital, Brisbane

The number of telehealth consultations per year ranged from 20 to 63 during the study period (Fig. 2a). Prior to 2010, there was a minimal annual variation with the average of 24 telehealth consultations per year. There was an increase in the annual number of telehealth consultations in the last three years of the study with 43 in 2011, 49 in 2012, and 63 in 2013 (47 % increase from 2011 to 2013). During the study period, the number of face-to-face nephrology consultations at the RCH in Brisbane steadily increased, averaging 622 (before 2010) and 791 (2010 to 2013) OPD appointments per year (27 % increase pre and post 2010) (Fig. 2b).

#### Telehealth activity by diagnosis

**Fig. 3:**
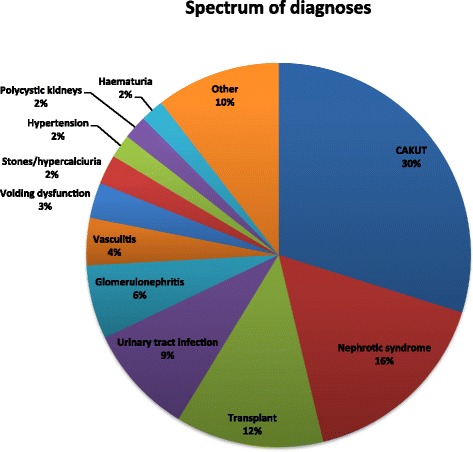
Spectrum of clinical diagnoses reviewed by paediatric nephrologists during telehealth consultations

Congenital anomalies of the kidney and urinary tract (CAKUT) were the largest group of renal diseases (30 %) involved in telehealth consultations, followed by nephrotic syndrome (16 %), kidney transplant (12 %), and urinary tract infection (9 %) (Fig. 3). The details of telehealth consultations (number, type and outcome) and patients (number, sex and age) in each diagnostic category are presented in Table 1. The Other group included diagnoses such as proteinuria, acute kidney injury, renal tubular acidosis, diabetes insipidus, and various syndromes (Bardet-Biedl, Denys-Drash, Williams, prune belly).

**Table 1:** Paediatric nephrology telehealth consultations by diagnosis

Diagnosis	Consults, *n*	Type of Consult	Outcome of Consult	Patients	Sex	Age, years median (range)
		N/R	TH/LOC/NS		M/F	
		*n*	*n*	*n*	*n*	
CAKUT	120	54/66	68/50/2	55	32/23	7 (3w – 18y)
Nephrotic Syndrome	66	24/42	45/20/1	29	20/9	7.5 (1y – 18y)
Kidney Transplant	50	4/46	46/4/0	6	6/0	12 (4y – 24y)
UTI	37	27/10	10/26/1	27	12/15	5 (6 m – 17y)
GN	25	14/11	14/11/0	14	9/5	12 (4y – 18y)
Vasculitis	16	4/12	11/5/0	6	1/5	15 (10y – 21y)
Voiding Dysfunction	12	10/2	3/9/0	11	3/8	8.5 (9 m – 17y)
Stones/Hypercalciuria	10	6/4	1/8/1	8	5/3	13 (2 m – 24y)
Hypertension	8	5/3	3/5/0	6	5/1	1.5 (10 m – 14y)
PKD	8	5/3	4/4/0	5	0/5	6.5 (3 m – 15y)
Haematuria	8	6/2	3/5/0	5	4/1	10.5 (2y – 15y)
Other	42	18/24	25/14/3	23	14/9	10 (6w – 21y)

*N* new, *R* review, *TH* telehealth follow-up, *LOC* local follow-up, *NS* not specified, *M* male, *F* female, *CAKUT* congenital anomalies of the kidney and urinary tract, *UTI* urinary tract infection, *GN* glomerulonephritis, *PKD* polycystic kidney disease, *w* weeks, *m* months, *y* years

#### Cost analysis

Assuming infrastructure costs for both the telehealth service and hospital outpatient service were already in place, the estimated annual cost of providing the paediatric nephrology telehealth service was $8,688 compared to $40,525 had the same sample of patients been seen in person at the RCH. This means an estimated cost-saving of $31,837 in 2013 ($505 saved per consultation) had patients travelled to the RCH for their appointment. The most substantial costs associated with the RCH outpatient service were patient and family travel and accommodation expenses, whereas participating specialist/paediatrician salaries were the highest costs associated with telehealth service (Table 2).

**Table 2:** Estimated costs of providing the paediatric telenephrology service by telemedicine and face-to-face consultations during a one-year period from January to December 2013 (63 consultations)

Cost	Telemedicine ($)	RCH Outpatients ($)
Telehealth coordinator ($38 per h), 33.33hrs^a^	1,266	0
Specialist ($140 per h), 22.22hrs^b^	3,111	3,111
Local admin support ($36 per h), 22.22hrs^c^	0	800
Regional presenter (paediatrician/medical officer) ($140 per h), 22.22hrs^b^	3,111	0
Regional admin support ($36 per h), 33.33hrs^b^	1,200	0
Patient travel^d^	0	29,294
Patient accommodation^d^	0	7,320
*Total Cost*	*8,688*	*40,525*

^a^University of Queensland hourly rates were used at Level 6 [22]. An additional 50 % of time was added to the number of hours allocated to telehealth coordinator to account for clinic preparation, such as booking regional or remote videoconference sites

^b^Specialist/Paediatrician hourly rates were calculated using the Queensland Governments Industrial agreement at the highest pay level (L29), plus a loading of 35 % for additional benefits [23]

^c^Local administration support hourly rate was calculated using the Queensland Health industrial agreements for Queensland Government Industrial agreement at a Level 4 with 21 % on costs [24]

^d^Queensland Health Patient Travel Subsidy Scheme [8]

## Discussion

In Australia, a country with a large geographical area, telemedicine plays an important role in the healthcare by improving access to specialist services while reducing the cost and inconvenience associated with traditional face-to-face consultations.

There is a limited published literature on the use of telemedicine in patients with renal disease, predominantly in adults with chronic kidney disease or end-stage kidney disease (ESKD). The reports indicate that telemedicine is a safe and effective modality of health care delivery in patients receiving peritoneal dialysis [9, 10] and haemodialysis [11, 12]. To our knowledge, there is only one published paediatric study reporting the use of online non real-time nephrology consultations for the patients and their families [13].

The results of our study show an increasing level of telehealth activity for paediatric nephrology consultations in Queensland, especially over the last three years. This is partially due to the addition of the third nephrologist to the service, but is also a reflection of the increased uptake by peripheral centres who recognize the advantages that telemedicine offers – access to specialist paediatric nephrology care from remote sites, convenience of staying home while receiving specialist care, and an educational opportunity for the regional medical teams. The introduction of Medicare payments for telehealth consultations by the Australian government from July 2011 might have also played a minor role in the increase of telemedicine activity over the last three years [14, 15].

With regard to the diagnosis, a wide spectrum of kidney problems have been involved in the telehealth consultations. The children with CAKUT comprised the majority of patients. Most of them were young children who remained in the telehealth follow up for many years. The second biggest group of patients were children with nephrotic syndrome, including those were steroid dependent and steroid resistant. Similarly to children with CAKUT, patients with nephrotic syndrome continued to receive local care with the advice/supervision provided by the specialist in Brisbane, with minimal travel to Brisbane.

A special group of renal patients are those with ESKD, either receiving dialysis treatment or the patients with kidney transplant. The medical care of these patients requires a high level of coordination and close follow up. Six children with kidney transplant who received 50 telehealth consultations were included in our study. All kidney transplants were performed by the kidney transplantation team in Brisbane. After the initial stabilization period (usually 3 months), patients with stable graft function were transferred to the care of local paediatricians, which allowed the regional healthcare team to deliver the care for these children under the guidance/supervision of the paediatric nephrologist in Brisbane. All kidney transplant recipients remained well for the duration of the study. We did not identify any patients on peritoneal dialysis in our audit. Historically, these patients have travelled to Brisbane for face-to-face appointments with a treating nephrologist. With increasing involvement in telehealth from local providers, there is an opportunity to use telemedicine in this group of patients.

Systematic review of more than 200 studies showed variable and inconclusive results with regard to cost-effectiveness of telemedicine [16]. Cost-minimisation studies in telehealth have shown substantial savings mainly due to potential reduction in travel costs for paediatric and adult services [17–20]. For the cost-saving analysis in the current study, we estimated the cost in 2013, the most recent year with the largest number of telehealth consultations. Our analysis shows substantial cost savings of telehealth consultations compared to face-to-face appointments of over $30,000 per year, each telehealth consultation being approximately $500 cheaper than traditional face-to-face visit in a tertiary hospital. Consistent with the finding of our previous reports, the majority of cost saving was related to patient/family travel and accommodation.

Telemedicine offers multiple advantages when compared to face-to-face appointments for people living far away from the hospital. Cost to the patients and their families in the form of travel, accommodation and out-of-pocket expenses is reduced. Cost saving to the health care system for patients requiring air travel and accommodation due to the distance from the tertiary hospital (most of the patients included in this study) or the multiple medical appointments is maximized. Educational benefits to the local teams are well documented [21]. Sharing of the medical information between the specialist team and the treating team providing the care to the patient is easier than the information exchanged through the infrequent letters and occasional telephone call. Direct communication minimizes misunderstanding and provides the space for the discussion and questions that might come up during the telehealth consultation. The presence of the child, family and both the local and supervising teams during the consultation improves the commitment of the family to follow the treatment plan and might improve adherence, as is demonstrated by the successful follow up of our patients after the kidney transplantation. The limitations of telemedicine include the difficulty establishing the rapport with new patient/family (more suitable for follow up than initial consultation) and impersonal nature of telemedicine, especially with significant changes involving the management of patients (initiation of dialysis, failing transplant).

## Conclusions

Our study demonstrates that paediatric telenephrology is a viable option of the long-term follow up of children, adolescents and young adults with various renal disorders, including those after kidney transplantation. Telehealth clinics offer multiple benefits to the patients, their families, treating teams, and the health care system. In the right circumstances, telehealth may be less expensive for the health service and offer greater access to a range of specialist services typically not available in regional and remote communities.

## Abbreviations

COH: Centre for Online Health
QTS: Queensland Telepaediatric Service
RCH: Royal Children’s Hospital
PTSS: Patient Travel Subsidy Scheme
CAKUT: Congenital anomalies of the kidney and urinary tract
ESKD: End-stage kidney disease
UTI: Urinary tract infection
GN: Glomerulonephritis
PKD: Polycystic kidney disease
